# Improved Neural Inductivity of Size-Controlled 3D Human Embryonic Stem Cells Using Magnetic Nanoparticles

**DOI:** 10.34133/bmr.0011

**Published:** 2024-03-15

**Authors:** Boram Son, Sora Park, Sungwoo Cho, Jeong Ah Kim, Seung-Ho Baek, Ki Hyun Yoo, Dongoh Han, Jinmyoung Joo, Hee Ho Park, Tai Hyun Park

**Affiliations:** ^1^ School of Chemical and Biological Engineering, Institute of Chemical Processes, Seoul National University, 1 Gwanak-ro, Gwanak-gu, Seoul 08826, Republic of Korea.; ^2^ Department of Bioengineering, Hanyang University, 222 Wangsimri-ro, Seongdong-gu, Seoul 04763, Republic of Korea.; ^3^ Center for Scientific Instrumentation, Korea Basic Science Institute, Cheongju, Chungbuk 28119, Republic of Korea.; ^4^ Center for Bio-based Chemistry, Korea Research Institute of Chemical Technology (KRICT), Ulsan 44429, Korea.; ^5^ SIMPLE Planet Inc., 48 Achasan-ro 17-gil, Seongdong-gu, Seoul 04799, Korea.; ^6^ Department of Biomedical Engineering, Ulsan National Institute of Science and Technology (UNIST), Ulsan 44919, Republic of Korea.; ^7^ Research Institute for Convergence of Basic Science, Hanyang University, Seoul 04763, Republic of Korea.; ^8^ Department of Nutritional Science and Food Management, Ewha Womans University, Seodaemun-gu, Seoul 03760, Republic of Korea.

## Abstract

**Background:** To improve the efficiency of neural development from human embryonic stem cells, human embryoid body (hEB) generation is vital through 3-dimensional formation. However, conventional approaches still have limitations: long-term cultivation and laborious steps for lineage determination. **Methods:** In this study, we controlled the size of hEBs for ectodermal lineage specification using cell-penetrating magnetic nanoparticles (MNPs), which resulted in reduced time required for initial neural induction. The magnetized cells were applied to concentrated magnetic force for magnet-derived multicellular organization. The uniformly sized hEBs were differentiated in neural induction medium (NIM) and suspended condition. This neurally induced MNP-hEBs were compared with other groups. **Results:** As a result, the uniformly sized MNP-hEBs in NIM showed significantly improved neural inductivity through morphological analysis and expression of neural markers. Signaling pathways of the accelerated neural induction were detected via expression of representative proteins; Wnt signaling, dopaminergic neuronal pathway, intercellular communications, and mechanotransduction. Consequently, we could shorten the time necessary for early neurogenesis, thereby enhancing the neural induction efficiency. **Conclusion:** Overall, this study suggests not only the importance of size regulation of hEBs at initial differentiation stage but also the efficacy of MNP-based neural induction method and stimulations for enhanced neural tissue regeneration.

## Introduction

Human embryonic stem cells (hESCs) have been a promising candidate in regenerative medicine and tissue engineering due to their pluripotency [1,2]. However, there have been limitations in clinical applications of the hESCs due to side effects such as tumor formation as a result of nonspecific differentiation [3,4]. To take full advantage of the hESCs, a strategy for precisely regulated differentiation of hESCs into targeted cell types has been required [3]. To improve the accuracy for lineage specification, various chemical cues have been used to differentiate hESCs, inducing designated signaling pathways [5–8]. Recently, in addition to such chemical factors, physical environment has been also considered a new key to control the hESC fate [9–11]. Therefore, generation of human embryoid bodies (hEBs), the 3-dimensional (3D) organization of hESCs, is suggested as a promising method to regulate the hESCs differentiation [12–14]. Since the hEBs are produced to mimic the biological niche during embryonic development, hESCs in the form of hEBs spontaneously lose self-renewal ability and differentiate [15,16].

In our previous studies, we developed high-throughput hEB generation and size control system, resulting in production of different sizes of hEBs: large hEBs that are 600 μm in diameter and small hEBs that are 150 μm in diameter [17]. Through the hEB generation method, uniformly sized hEBs were efficiently generated without Rho-associated protein kinase inhibitor. Then, the effect of hEB size on differentiation into specific germ layers was detected. We suggested the proper size for each germinal layer: 150 μm for ectoderm and 600 μm for endoderm and mesoderm. The significance of hEB size on directing the hESC lineage determination was measured.

There have been several approaches to control the size of 3D cell aggregates. First, there have been conventional methods for cell aggregate preparation [18–22]. The 3D cell aggregates were generated using nonadhesive plates, round-bottom well plates, hanging drops, pellets, and rotating incubators. The advantages of those conventional 3D cultivation are rapid and scalable cultivation; however, controlling the size of cell aggregates was difficult, resulting in broad size distribution. Therefore, 2-dimensional (2D) hydrogel surface-based manners [23–28] and 3D hydrogel embedding-based manners [29–37] were developed for precise size regulation. Using the 2D and 3D hydrogel-based cell aggregate generation methods, the maximum 3D cell aggregate diameter was determined by the size of the coated well. However, in many cases, 2 or more aggregates were observed in a well, resulting in failure of precise cell aggregate size control. Nevertheless, the advantages of those 2D and 3D hydrogel-based manners are well-defined 3D cell aggregate generation compared with the conventional methods. In addition, cell viability and function were increased which was decreased in conventional methods due to shear stress. Recently, there are several technology-assisted preparations of cell aggregates [38]: Cell-incorporating magnetic nanoparticles (MNPs) and external magnetic field-based method [39,40]; electrical force-driven aggregate generation [41]; high cell density within 3D hydrogels [42,43]; microfluidic devices [44,45]; and microgels using aggregation manner [46]. Since those multicellular organizations have been mainly applied for drug discovery or tissue engineering, various types of tissue-derived somatic cells or cell lines such as liver cells, kidney cells, and pancreatic cells have been used instead of the hESCs for 3D cell aggregates formation. Therefore, technologies have been developed with a focus on realizing and providing an appropriate physical environment for tissue realization, rather than focusing on developing factors such as sophisticated size control required for lineage specification of the hEBs. In this study, however, we aimed to direct the initial differentiation and consequently improve the efficiency of neural induction by using a technique to regulate the size of hEB from the generation stage.

In this study, for size-controlled hEB generation, MNPs were used, which were isolated from the magnetic bacterium, *Magnetospirillum* sp. AMB-1. The characteristics of MNPs have been analyzed in previous related studies [9,17,47–50]. Optimization of culture conditions for magnetotactic bacterium, AMB-1, which produces MNPs, and the resulting growth curve and MNPs production efficiency have been confirmed [50]. Also, the characteristics such as morphology and zeta potential of the bacteria-derived MNPs have been analyzed [48]. Furthermore, the strength of the magnetic force on the pinpoint was measured in a concentrated magnetic force system, and the behavior of the magnetized mammalian cells by the external magnetic force was observed in real time [47]. The aspects of MNPs incorporated into stem cells were observed by a transmission electron microscope (TEM), and cell viability and the amount of MNPs in a cell depending on the MNP concentration were analyzed [9,49]. Furthermore, the morphology of MNPs introduced into ESCs was observed under on-feeder and feeder-free conditions, respectively [17]. According to those former results, magnetic bacteria were cultured under anaerobic conditions and the MNPs were synthesized within the bacterial cytoplasm [47,50], exhibiting high intracellular delivery efficiency with low cytotoxicity [9,17,47–50]. Since the MNPs possess lipid bilayer of the host bacteria, the particles were readily endocytosed by mammalian cells including stem cells, resulting in magnetization of the cells. The morphology of the MNPs were observed through a scanning electron microscope (SEM) in Fig. S1, and intracellularly delivered MNPs were also observed through TEM in Fig. S2. Therefore, the MNPs were considered to be a useful tool for simultaneous mechanical stimulation across various forces such as static magnetic field and magnet-derived shear stress [9,51]. Those magnetic force-induced biophysical stimulations were applied to enhance tissue-specific differentiation of stem cells [17,52].

**Fig. 1.**
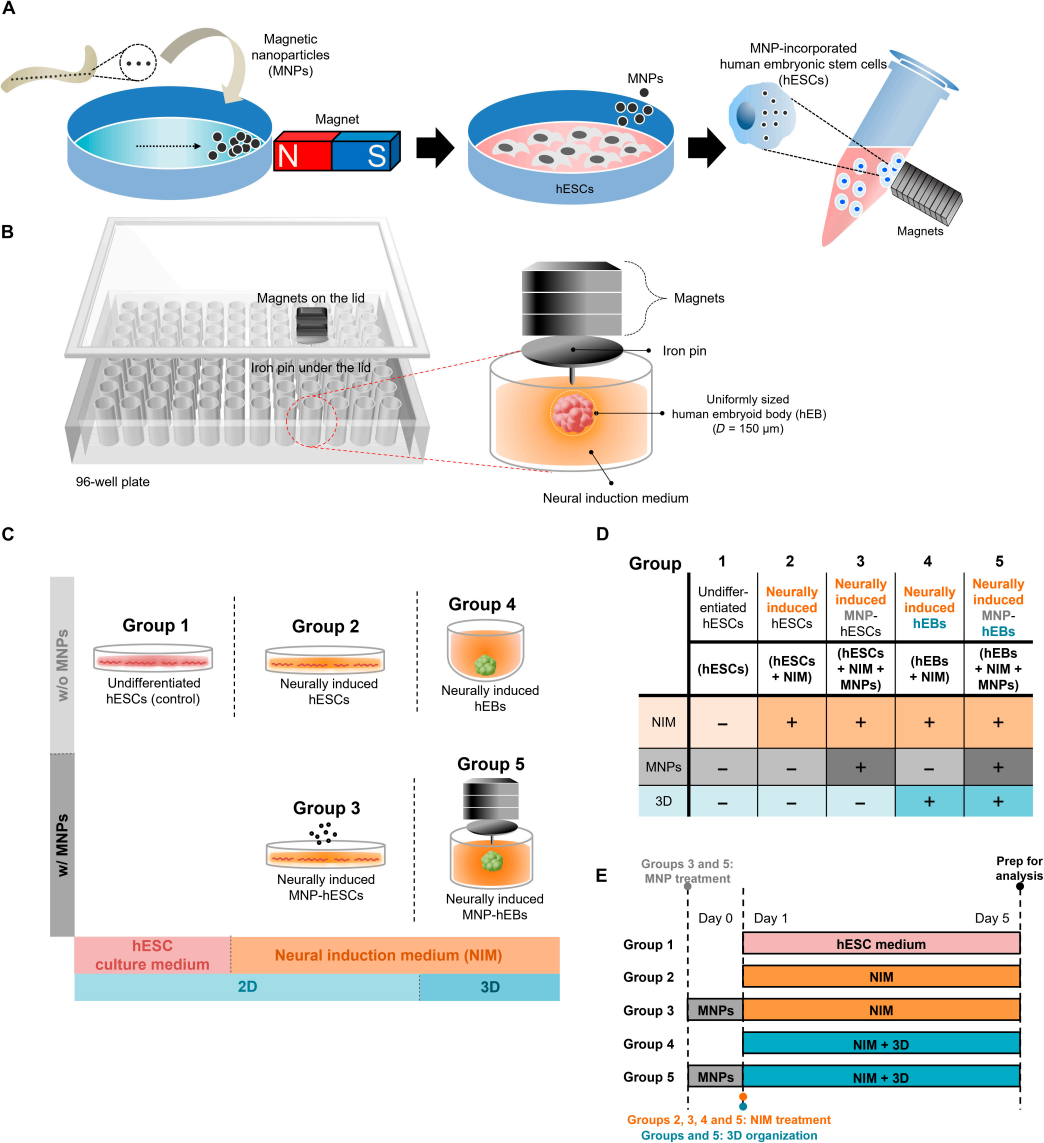
Schematics of MNP-based hEB generation method and designation of experimental groups. (A) Overall steps of hESC magnetization. After ultrasonic disruption of magnetic bacteria, MNPs were isolated with neodymium magnets. The hESCs were treated with collected MNPs, and MNP-incorporated hESCs were separated using magnets. (B) Schematics for concentrated magnetic force system and neural induction of hEBs. To efficiently differentiate the hESCs into neural commitments, a high-throughput method utilizing the MNPs and concentrated magnetic force system in a 96-well plate was applied. Therefore, uniformly sized hEBs (150 μm in diameter) were produced. (C) Illustrations of experimental groups: Undifferentiated pluripotent hESCs as the control group (group 1); neurally differentiated hESCs (group 2); neurally induced hESCs with MNPs (group 3); neurally induced hEBs, generated in the noncoated round-bottom plates (group 4); neurally induced MNP-incorporated hEBs (group 5). (D) Each experimental group was decided by the existence of 3 factors. Firstly, NIM instead of hESC culture medium. Secondly, MNPs. Lastly, 3D cultivation environment. (E) Timeline of each experiment. MNPs were treated to groups 3 and 5 on day 0. In neurally induced groups (groups 2 to 5), NIM were treated on day 1 and all the cells were analyzed on day 5. For 3D organization, cells were resuspended through NIM in groups 4 and 5.

In this study, uniformly sized MNP-hEBs (150 μm in diameter) were generated using concentrated magnetic force and the MNPs and then neurally differentiated in neural induction medium (NIM) (Fig. 1). In order to investigate the improvement of hESC neural inductivity in the form of MNP-hEBs, experimental groups were compared based on addition of 3 variables for neural differentiation: NIM, MNPs, and 3D. Since the hEBs mimic the stiffness of the native tissue [53,54], uniformly sized hEB generation is considered to be a worthwhile strategy to facilitate neuronal differentiation, providing a similar physical environment to brain tissue. In addition, MNP-hEB production could be an efficient approach enhancing cell-to-cell interactions through magnetic force, resulting in improved neural induction.

## Materials and Methods

### Preparation of MNPs

MNPs (Fe_3_O_4_), obtained from an anaerobically cultured magnetotactic bacterium (*Magnetospirillum* sp. AMB-1, American Type Culture Collection), were used to generate 3D hESCs, called hEBs. The magnetic bacterium was cultured in a modified magnetic spirillum growth medium, as previously described [48,50]. In brief, bacteria were cultivated via a fermenter (FMT-ST-S05, Fermentec) in an anaerobic condition for 5 d at 27 °C. Then, the bacterial cells were centrifuged at 11,300 × g for 20 min, and then the collected cells were sonicated with 35% amplification for 15 min (VCX500, Sonics & Materials). The MNPs were isolated from bacterial cell debris using 100-mm petri dishes with neodymium-iron-boron (NdFeB) magnets attached below. Only MNPs, except for cell debris, were attached along the perimeter of the magnets. Therefore, the MNPs were collected while the cell debris was washed out. The separated MNPs were washed 3 times using phosphate-buffered saline (PBS; Welgene), relying on magnetic adhesion. Then, the MNPs were sterilized using an autoclave. After measuring the concentration of iron ions using inductively coupled plasma atomic emission spectroscopy (ICPS-7500, Shimadzu), the MNPs were stored in a concentration of 1 mg/ml in PBS at 4 °C. Before use, the MNPs were dispersed using bath type ultrasonicator (JAC 1002, Kodo Technical Research) for 10 min.

### Observation of MNPs

In order to investigate the morphological characteristics of the MNPs, SEM analysis was performed. After dispersed in PBS, droplets of MNPs aqueous solution were placed on a 12-mm circular coverslip, lyophilized using a freeze dryer (Hanil, Seoul, Korea), and then sputter-coated with the platinum. The SEM images were obtained using a field emission SEM (JSM-6700F, JEOL, Japan). The MNPs endocytosed in hESCs were observed through TEM. After incubation with 20 μg ml^−1^ cell-penetrating MNPs, the hESCs were fixed with paraformaldehyde-glutaraldehyde solution (Karnovsky’s Fixative) for 2 h at 4 °C. The cells were then washed with 0.05 M sodium cacodylate buffer. Subsequently, the cells were fixed with 2% osmium tetroxide with 0.1 M cacodylate buffer for 2 h and washed using distilled water, followed by overnight 0.5% uranyl acetate treatment for negative staining at 4 °C. After serial dehydration with sequentially concentrated ethanol from 30% to 100%, the cells were treated with propylene oxide to remove the residual ethanol. Finally, they were penetrated by propylene oxide with resin mixture and were then embedded in resin. The embedded samples were cut using an ultramicrotome (EM UC7, Leica, Germany) and were then observed via TEM (JEM1010, JEOL, Japan).

### Culture of hESCs

hESCs (SNUhES31) were supplied in passage 23 from the Seoul National University Medical Research Center upon approval from the Seoul National University Institutional Review Board (IRB No.1402/002-006). hESCs were maintained in standard hESC growth condition and sustained their pluripotency, following previously described protocols [2,55]. Briefly, the hESCs were cultured with STO mouse fibroblast cells (STO cells), which were mitotically inactivated by mitomycin C (Sigma), on 0.2% gelatin-coated cell culture dishes in Dulbecco's Modified Eagle Medium with Nutrient Mixture F-12 (DMEM / F-12, Gibco) supplemented with 20% KnockOut Serum Replacement (Gibco), 4 ng/ml basic fibroblast growth factor (Invitrogen), 0.1 mM β-mercaptoethanol (Sigma), 0.1 mM nonessential amino acids (Gibco) and 50 units/ml penicillin and 50 μg/ml streptomycin (Gibco). For subculture, the hESC colonies were disassembled by modified Pasteur pipettes and replated on new dishes with a fresh STO feeder layer, every 5 to 7 d. The medium of hESCs was daily replaced. The passages of hESCs utilized in this study were between 32 to 42.

For feeder-free culture, hESCs were transferred to dishes coated with Geltrex (Gibco), and their culture medium was switched to Essential 8 medium (Gibco) without any influences on pluripotency [15]. Cells were cultivated at 37 °C with 5% CO_2_ in a humidified incubator.

### Generation of hEBs and differentiation of 2D and 3D hESCs

For generation of hEBs without MNPs, 96-well noncoated round-bottom plate (SPL) was coated with 0.1% F-Pluronic 127 (Sigma) in double distilled water for 1 h. Feeder-free hESCs were detached with accutase (Millipore), and 1 × 10^4^ cells were added to a well in order to generate hEBs without MNPs (hEBs + NIM).

For generation of hEBs with MNPs, the feeder-free hESCs were treated with 20 μg/ml of MNPs for 24 h. The MNP-incorporated hESCs (MNP-hESCs) were detached with accutase and then sufficiently magnetized hESCs were isolated by the NdFeB magnets for 1 min. After separation of MNP-hESCs, the suspended cells were applied to a concentrated magnetic force system, which was manufactured as described in the previous work [47]. In brief, the lids of 96-well plates were prepared with NdFeB magnets (10 mm × 5 mm × 6 mm) placed upon the lids, and iron pins attached to the magnets under the lids. A total of 1 × 10^4^ cells in a volume of 130 to 135 μl were added to each well (the volume depends on the depth of iron pin). Then the magnetized hESCs were gathered toward the iron pin at which magnetic force was concentrated, resulting in generation of uniformly sized MNP-hEBs (150 μm in diameter) underneath the medium surface (hEBs+ NIM + MNPs).

For initial neural differentiation, 2D and 3D hESCs were cultured in NIM (Gibco, 21103049) for 5 d. The medium was changed on the third day. Cells were cultured at 37 °C with 5% CO_2_ in a humidified incubator.

### Quantified reverse transcription-polymerase chain reaction

In order to investigate neural inductivity analyzing mRNA expression, quantified reverse transcription–polymerase chain reaction (qRT-PCR) was conducted. Total RNA was extracted with TRIzol RNA Isolation Reagents (Invitrogen), according to the manufacturer’s instructions [16]. After centrifugation, the collected cells were lysed with TRIzol solution and then incubated for 5 min at room temperature. Chloroform (Millipore) was added at 20% (v/v), and the total solution was shaken for 15 s, then subsequently incubated for 3 min at room temperature. After 12,000 × g centrifugation for 15 min at 4 °C, the only upper colorless aqueous phase was isolated to a fresh tube. Then, isopropyl alcohol (Millipore) was added at 1:1 volume, and the whole solution was centrifuged at 12,000 × g for 10 min at 4 °C. After centrifugation, the supernatant was removed, and then RNA was washed with 75% ethanol (Millipore) through a vortex mixer. After centrifugation at 7,500 × g for 5 min at 4 °C, the RNA pellet was dried for 1 h at room temperature and dissolved in RNase-free water (iNtRON Biotechnology), subsequently heat-treated at 55 to 60 °C for 10 min. For reverse transcription, 500 ng of total RNA was applied to a Moloney murine leukemia virus cDNA synthesis kit (Enzynomics), following manufacturer’s instructions. The qRT-PCR was carried out with TOPreal qPCR 2X PreMIX (Enzynomics), utilizing a StepOnePlus Real-Time PCR System (Applied Biosystems). As a pluripotency marker gene, octamer-binding transcription factor 4 (*OCT4*) was used. For evaluation of neural induction, growth-associated protein 43 (*GAP43*), β3-tubulin (*TUBB3*), nestin (*NES*), and glial fibrillary acidic protein (*GFAP*) were utilized as neuronal marker genes. glyceraldehyde 3-phosphate dehydrogenase (*GAPDH*) was used as a reference, housekeeping gene. For qRT-PCR, all the experiments were conducted in at least 3 replicates. The expression level of each gene was normalized to that of the housekeeping gene, *GAPDH*. The relative fold change in a pluripotency marker gene expression level and the neural marker gene expression levels compared to that of the control group (undifferentiated hESCs) was represented in a graph. All data were expressed as the mean ± standard deviation. Statistical significance was determined using SigmaPlot software. The Student *t* test was used to compare 2 groups. The statistical significance was represented by * for *P* < 0.05, ** for *P* < 0.01, and *** for *P* < 0.001.

### Immunocytochemical analysis

In order to investigate initial neural differentiation, immunocytochemical analysis was conducted. The hEBs were also observed in a 2D attached state in order to confirm the single cellular morphology as in 2D hESCs. Therefore, cell migration from the hEBs to surroundings was also observed. Cells were fixed with 4% paraformaldehyde diluted in PBS for 10 min at room temperature [56–58]. Then, the fixed cells were permeabilized with 0.25% Triton X-100 diluted in PBS (PBST) for 10 min, and they were blocked with 3% bovine serum albumin (BSA) in 0.1% PBST for 1 h. After the fixation, permeabilization, and blocking in series, cells were incubated with primary antibodies diluted to final concentration of 1:1,000 in 1% BSA in 0.1% PBST solution overnight on a rocker at 4 °C. To investigate pluripotency, anti-NANOG antibody (Cell Signaling Technology, D73G4), and anti-sex determining region Y-box 2 (SOX2) antibody (Cell Signaling Technology, D6D9) were used. For the detection of neural induction, anti-glial fibrillary acidic protein (GFAP) antibody (Abcam, ab10062), anti-paired box 6 (PAX6) antibody (Abcam, ab5790), and anti-prospero homeobox protein 1 (PROX1) antibody (Abcam, ab101851) were used. To observe related mechanisms of the accelerated neural induction, anti-glial cell-line-derived neurotrophic factor (GDNF) antibody (Abcam, ab18956), anti-neural cell adhesion molecule (NCAM) antibody (Cell Signaling Technology, 3606S), anti-microtubule-associated protein 2 (MAP2) antibody (Abcam, ab32454), and anti-FAK antibody (Abcam, ab81298) were used. After incubation with the primary antibodies, cells were washed with PBST, and then they were incubated with the secondary antibodies, diluted in 5% BSA in PBST at 1:1,000 dilution, for 1 h at room temperature. As secondary antibodies, an anti-rabbit immunoglobulin G (IgG)-–Alexa 488 conjugate antibody (Invitrogen, A11008), an anti-rabbit IgG-Alexa 594 conjugate antibody (Invitrogen, A11032), and an anti-mouse IgG-Alexa 594 conjugate antibody (Invitrogen, A11037) were utilized. Lastly, the cells were incubated with 4',6-diamidino-2-phenylindolefor 5 min in the dark and then observed by confocal laser scanning microscopy (Leica).

### Western blotting

To detect expression of neural-induction-related mechanism proteins, western blotting was performed. After 1,200 × g centrifugation for 5 min, 3 × 10^5^ cells were resuspended in RIPA Buffer (LPS Solution) with protease inhibitor cocktail (Abcam), at 1:100 dilution. The cells were lysed for 15 min at room temperature, and then the lysates were incubated at 100 °C for 5 min in 2 mM EDTA with sulfate-polyacrylamide gel electrophoresis protein loading buffer (Intron Biotechnology). Then 20 μl of samples were loaded to each well of 10% sulfate-polyacrylamide gel electrophoresis gel, and separated by electrophoresis. After transferring to nitrocellulose blotting membranes (GE Healthcare Life science) using a Trans-Blot SD Semi-Dry Transfer Cell (Bio-Rad, USA), the transferred membrane was blocked with 5% BSA in 0.1% PBST for 1 h and then incubated with primary antibody diluted in 1% BSA in 0.1% PBST (1:1,000 dilution) at 4 °C, overnight. After incubation with horseradish peroxidase-conjugated secondary antibody diluted in 5% BSA in 0.1% PBST at 1:2,000 dilution for 1 h, the membrane was treated with Luminata Western HRP Chemiluminescence Substrates (Millipore) and signals were measured with G: BOX Chemi XL system (Syngene). The primary antibodies used for western blotting were Wnt3 antibody (Abcam, ab32249), Wnt5α antibody (Abcam, ab72583), and GDNF antibody (Abcam), NCAM antibody (Cell Signaling Technology), MAP2 antibody (Abcam), and FAK antibody (Abcam). As a reference protein, the β-actin antibody (Abcam, ab8227) was used.

### Statistical analysis

All the experiments were conducted in at least 3 replicates. All data were expressed as the mean ± standard deviation. Statistical significance was determined using SigmaPlot software. The Student *t* test was used to compare 2 groups. For all experiments, statistical significance was represented by * for *P* < 0.05, ** for *P* < 0.01, and *** for *P* < 0.001. For morphological analysis, at least 150 random cells for each experimental group were analyzed. Each measured value was divided by the number of cells. Thus, the value was expressed as the value per cell.

## Results

### Neural induction of MNP-incorporated hEBs

In this study, neural inductivity of the hESCs was investigated depending on variables of 3 factors: NIM, MNPs, and 3D culture condition. For the initial neural differentiation of 2D and 3D hESCs, the hESCs were cultured in NIM instead of hESC culture medium for 5 d, except for the control group (undifferentiated hESCs; group 1). Then, the MNPs were used for enhanced neural induction, providing 3D organization of the hESCs. Therefore, after isolation from disrupted magnetic bacteria, the MNPs were applied to hESCs and sufficiently magnetized hESCs were separated using static magnets (Fig. 1A). In order to generate MNP-hEBs, the MNP-incorporated hESCs were added to concentrated magnetic force system, in which magnetic force of the magnets on the lid was focused at the pinpoint of iron pin under the lid (Fig. 1B) [47]. Thus, those magnetized hESCs were gathered at the point of concentrated magnetic force, resulting in generation of 3D hESCs under the surface. Furthermore, the diameter of the generated hEBs was uniformly regulated by adjusting the number of hESCs applied to a well (Fig. S3). Therefore, using the MNP-based hEB generation method, uniform hEBs (150 μm in diameter) were manufactured readily, and neural inductivity of MNP-hEBs (hEBs + NIM + MNPs; group 5) was investigated comparing with other experimental groups as follows: undifferentiated hESCs as control group (hESCs; group 1), conventionally differentiated hESCs with NIM (hESCs + NIM; group 2), neurally induced hESCs with NIM and MNPs (hESCs + NIM + MNPs; group 3), and conventionally obtained and differentiated hEBs in NIM (hEBs + NIM; group 4) (Fig. 1C and D). When the magnetized hESCs were added to a well of 96-well plates without the concentrated magnetic force system, some of the cells were suspended on surface and the others were sunken on the bottom, resulting in failure of hEB formation (Fig. S4).

### Morphological analysis of neurally induced hESCs

**Fig. 2.**
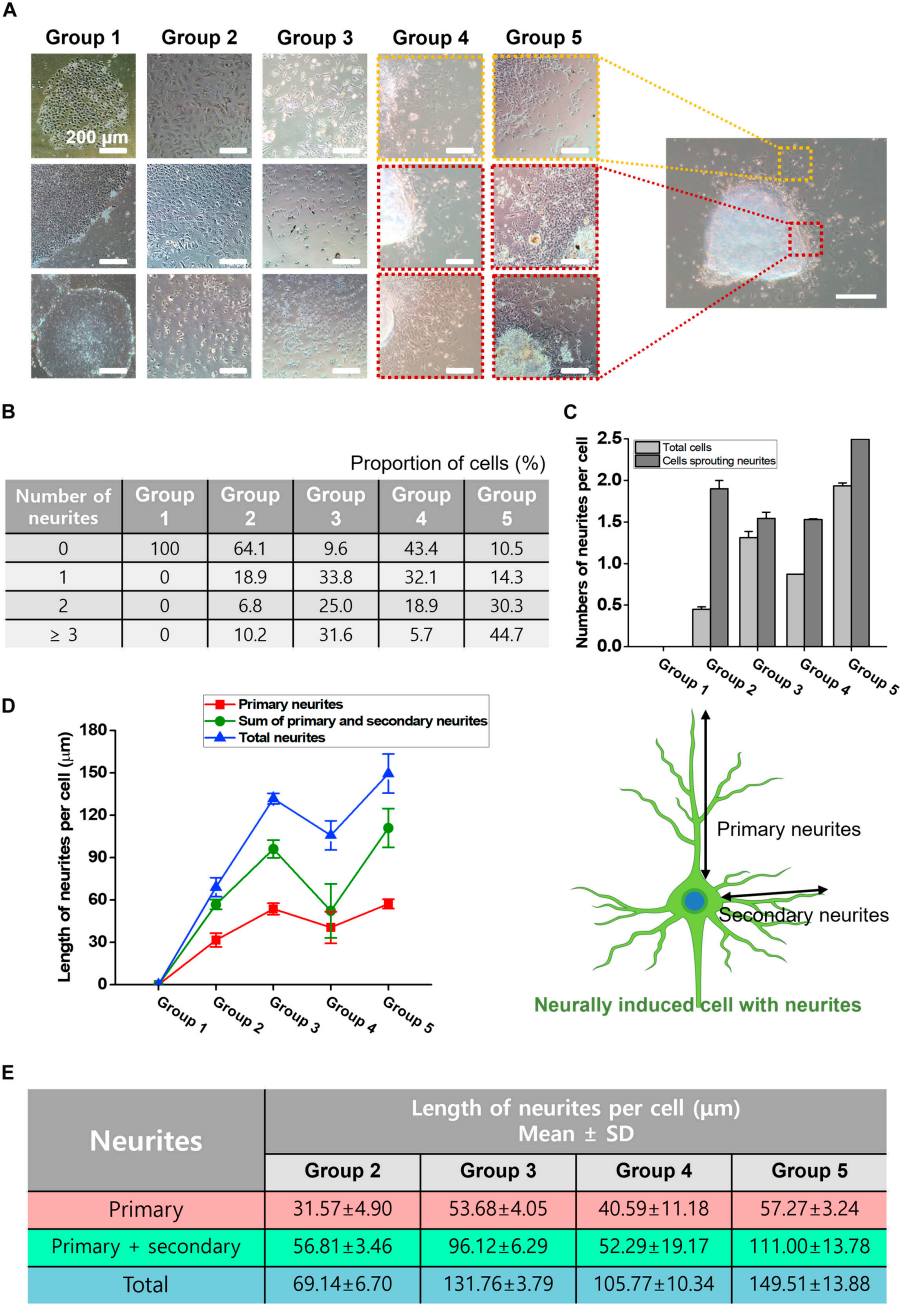
Morphological analysis of neurally induced 2D and 3D hESCs. (A) Microscopic images of hESCs in experimental groups. All the differentiated hESCs were neurally induced with NIM for 5 d. After 5-d-long differentiation, the hEBs were also observed in a 2D attached state in order to confirm the single cellular morphology as in 2D hESCs. Therefore, cell migration from the hEBs to surroundings was also observed. Yellow dotted squares indicate migrated cells from the 3D aggregates. Red dotted squares indicate the edge of hEBs, including 3D aggregates and migrated cells. (B and C) Number of neurites per cell. (B) The proportion of cells (%) according to the number of neurites in all experimental groups. (C) The number of neurites in total cells, including the cells without neurites, was demonstrated as black bars, and the number of neurites in only cells sprouting neurites was represented as gray bars. (D and E) Length of neurites per cell. (D) The length of primary neurites (red line), the sum of primary and secondary neurites (green line), and sum of total neurites (blue line) were investigated, respectively. (E) Mean values of neurite length. Scale bars, 200 μm. For morphological analysis, at least 150 random cells for each experimental group were analyzed.

Shape of the hESCs was observed to validate neural differentiation (Fig. 2A). In the control group (group 1), typical morphology of pluripotent hESCs was observed. The cells presented a round-shaped appearance, in compact colonies formed by the intact hESCs. Otherwise, in neurally induced hESCs (group 2), not only round cells but also angular cells coexisted. Also, those angular cells were observed where the cell density was low, far from the center of colonies. In neurally induced MNP-hESCs (group 3), neurally induced hEBs (group 4), and neurally induced MNP-hEBs (group 5), a larger number of angular cells were observed, and prominent neurite protrusions were shown. Several neurites sprouted from one cell, and some of those neurites were significantly longer.

To quantify the cell morphology related to neural induction, number of neurites per cell was examined (Fig. 2B and C). In group 1, cells remained spherical, resulting in the absence of neurite-extending cells, whereas all the other groups (group 2 to 5) expressed angular-shaped cells with neurites. The ratio of cells without neurites significantly decreased, while the proportion of cells with neurites increased according to neural induction with 3 variables (NIM, MNPs, and 3D). In particular, not only the ratio of cells with more than one neurite but also the average number of neurites per cell has been remarkably increased in group 5.

Also, the length of neurites was calculated as follows: length of primary neurites (the longest neurite in a cell), summarized length of primary and secondary neurites (the longest neurite + the second longest neurite in a cell), and summarized length of total neurites from each cell (Fig. 2D and E). As a result, the length of primary neurites (red line in Fig. 2D) and summarized length of primary and secondary neurites (green line in Fig. 2D) significantly increased in MNP-treated groups (groups 3 and 5) compared with the conventionally differentiated hESCs (group 2) (*P* < 0.001, respectively). Regarding the length of total neurites from one cell, the value remarkably increased in group 3 and both types of hEBs (groups 4 and 5), compared to group 2 (blue line in Fig. 2D; *P* < 0.001).

### Genetical analysis of neural induction marker genes

**Fig. 3.**
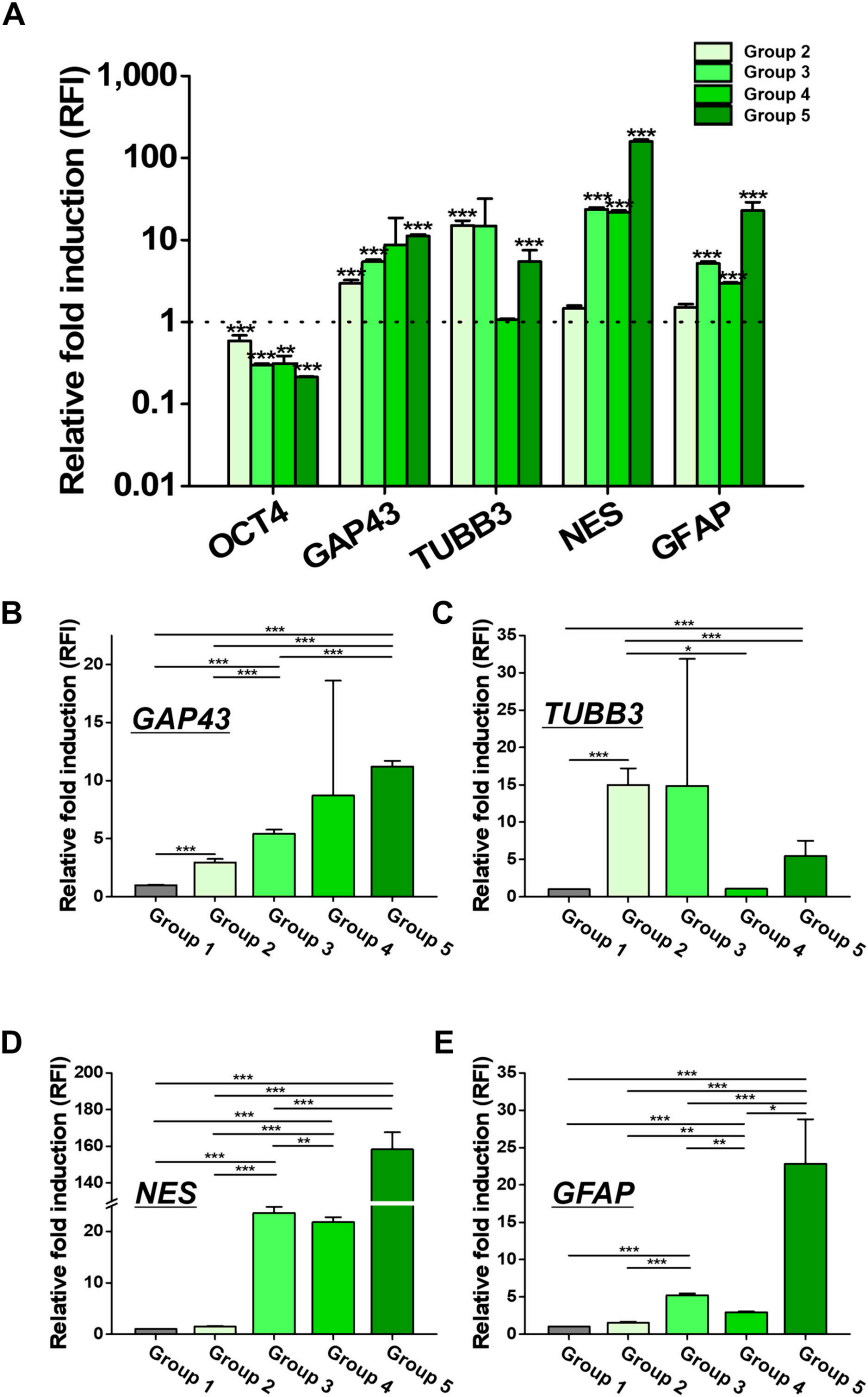
Genetical analysis of a pluripotency marker gene and neural induction marker genes. (A) Relative expression of mRNA in all the experimental groups. The expression level of each gene was normalized to that of the housekeeping gene, *GAPDH*. The relative fold-change value of each gene compared to the expression level of control was represented in the graphs (group 1, undifferentiated hESCs = 1). Expression of *GAP43* (B), *TUBB3* (C), *NES* (D), and *GFAP* (E) was compared among the groups. *P < 0.05, **P < 0.01, and ***P < 0.001.

To investigate neural induction, genetical analysis was performed, measuring mRNA expression levels (Fig. 3). According to Fig. 3A, the expression of *OCT4*, a pluripotency marker [59], statistically decreased in neurally induced 2D and 3D hESCs (group 2 to 5), compared with the value of control group (group 1, horizontal line indicating 1).

In Fig. 3B, expression of *GAP43* was compared, and thus neuronal growth and neurite formation were investigated [60]. Statistically significant increase was observed depending upon addition of variables for neural induction (NIM, MNPs, and 3D; sequentially from groups 1 to 5). Therefore, relative fold induction values remarkably increased in group 2 (neurally induced hESCs) comparing with group 1, also in group 3 (neurally induced MNP-hESCs) than group 2 (neurally induced hESCs), finally in group 5 (neurally induced MNP-hEBs) than group 3 (neurally induced MNP-hESCs) (*P* < 0.001).

Expression of *TUBB3* was compared in Fig. 3C, which indicates microtubule formation of early committed neurons [61]. The relative value was up-regulated in groups 2 and 3, compared with not only group 1 but also groups 4 and 5. Therefore, neurally induced 2D hESCs in attached condition (groups 2 and 3) showed enhanced expression of *TUBB3* compared with neurally induced 3D hESCs in suspended condition (groups 4 and 5).

Regarding *NES*, a specific marker of neural stem cells related to the growth of intermediate filament and axon of neural-precursor cells [9], the expression level significantly increased in group 3 and neurally induced hEBs regardless of MNP existence (groups 4 and 5), compared with groups 1 and 2 (*P* < 0.001, respectively) (Fig. 3D).

Similar tendency was observed in expression of *GFAP*, an astrocyte marker indicating intermediate filament growth and cell morphology maintenance (Fig. 3E). Expression of *GFAP* mostly increased in group 5, comparing to the other experimental groups.

### Immunocytochemical analysis of pluripotency and neural induction marker proteins

**Fig. 4.**
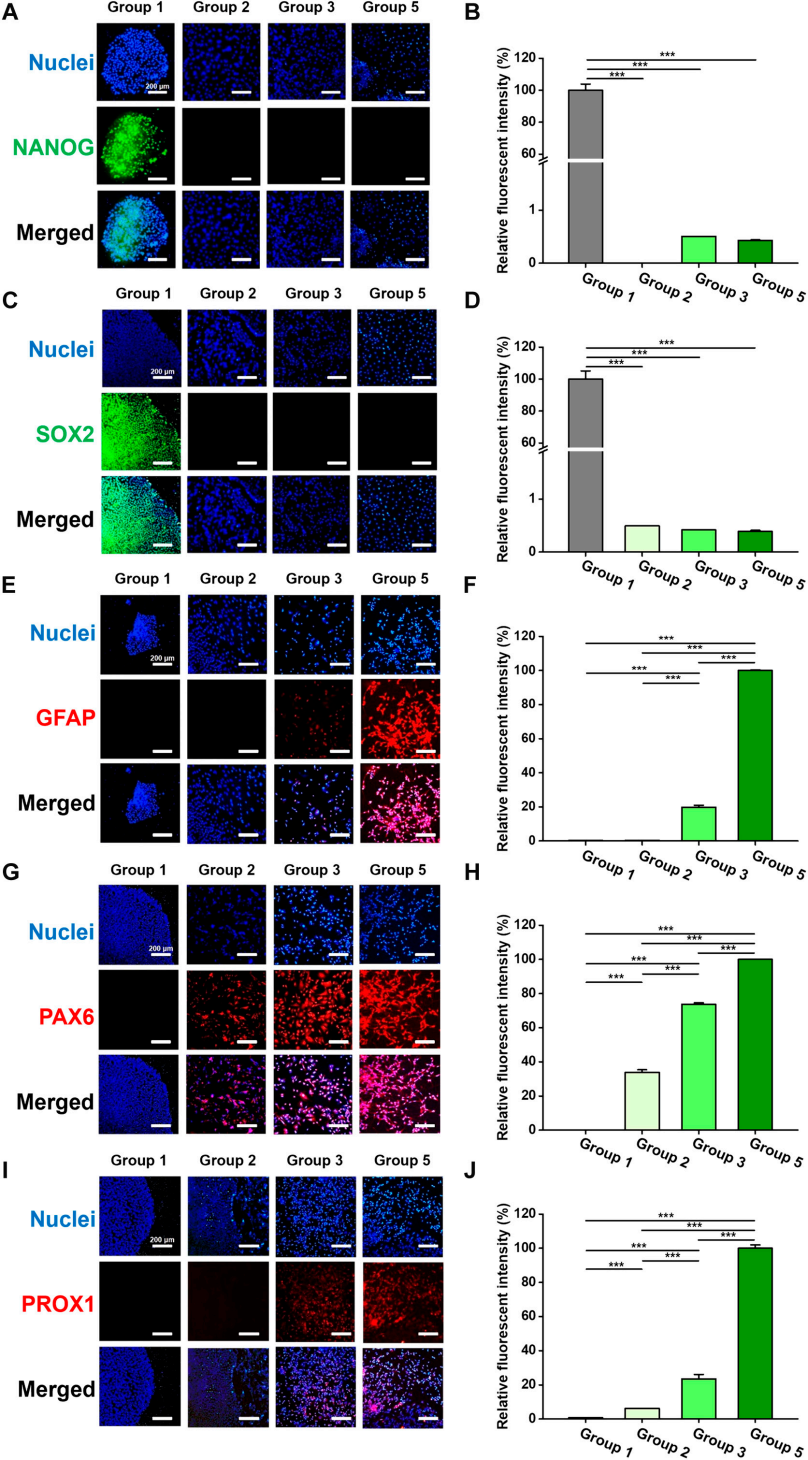
Immunocytochemical analysis of pluripotency marker proteins and neural induction marker proteins. (A and C) Immunocytochemistry of pluripotency marker proteins. Nuclei are represented as blue, and pluripotency marker proteins, NANOG (A) and SOX2 (B), are shown as green. Only in group 1, both NANOG and SOX2 were obviously expressed contrary to the other groups. (B and D) Quantified fluorescence intensity of pluripotency marker proteins. The expression level of pluripotency marker proteins was quantified relative to group 1 (100%), which showed maximum expression in both NANOG (B) and SOX2 (D). The relative expression of NANOG and SOX2 in neurally induced 2D and 3D hESCs was statistically down-regulated. (E, G, and I) Immunocytochemistry of neural induction marker proteins. Nuclei were represented as blue and neural induction marker proteins, GFAP (E), PAX6 (G), and PROX1 (I) are shown as red. The expression of those maker proteins showed an increasing tendency toward the end group. (F, H, and J) Quantified fluorescence intensity of the neural induction marker proteins. The expression level of neural induction marker proteins was quantified relative to group 5 (100%), which showed maximum expression in GFAP (F), PAX6 (H), and PROX1 (J), respectively. All the differentiated hESCs were neurally induced with NIM for 5 d. After 5-d-long differentiation, the hEBs were also observed in a 2D attached state in order to confirm the single cellular morphology as in 2D hESCs. Therefore, cell migration from the hEBs to surroundings was also observed. Scale bars, 200 μm. ***P < 0.001.

To analyze and compare the neural inductivity, expression of pluripotency proteins and several neural induction-related proteins was investigated through immunocytochemical analysis (Fig. 4). As representative pluripotency marker proteins, NANOG and SOX2 were used. The nuclei of the cells were labeled as blue, and the pluripotency markers, NANOG and SOX2, were labeled as green (Fig. 4A and C). According to the microscopic fluorescence images, the expression of NANOG and SOX was observed only in the control (group 1), while there was not obvious expression of the pluripotency proteins in the other groups (neurally induced 2D and 3D hESCs; group 2 to 5). Also, the nuclei shown as blue in group 1 represented the cells in compact colonies, where the undifferentiated pluripotent hESCs existed intactly. As a result, cells lost pluripotency and intercellular distance increased, resulting in the loss of colony form. Then, fluorescence intensity was quantified, and the calculated values were compared among the groups (Fig. 4B and D). The expression of pluripotency markers was normalized by the value of group 1, which expressed the highest intensity (100%). Compared to group 1, the relative intensity values of NANOG and SOX2 were statistically decreased (*P* < 0.001), remaining under 0.5%.

As neural induction marker proteins, GFAP, PAX6, and PROX1 were used: GFAP represents the growth of intermediate filament and the maintenance of cellular conformation of astrocytes [62–65]; PAX6 is a regulatory transcription factor for neurogenesis and brain development during embryogenesis; PROX1 indicates the development of white matter of brain during embryogenesis [66]. For evaluation of the neural induction marker proteins, nuclei and marker proteins were labeled as blue and red, respectively (Fig. 4E to J). In Fig. 4E and I, the red fluorescence indicating GFAP and PROX1 was not observed in groups 1 and 2, while in MNP-treated groups (groups 3 and 5), GFAP and PROX1 was expressed. In addition, the expression of red fluorescence was remarkably enhanced in group 5. Therefore, the expression level of GFAP and PROX1 was normalized by the value of group 5, which showed the maximum expression in both of GFAP and PROX1 (100%) (Fig. 4F and J). In Fig. 4F, the relative intensity values of GFAP in groups without MNPs (groups 1 and 2) were both under 1.0% and in group 3 was 19.8%. In Fig. 4J, expression value of PROX1 was under 1.0% in group 1 and 6.1% in group 2, respectively, and the relative intensity was 23.5% in group 3.

Regarding the expression of PAX6, group 1 did not show any red fluorescence indicating PAX6, and neurally induced 2D and 3D hESCs (groups 2, 3, and 5) showed distinct PAX6 expressions and the intensity of red fluorescence was enhanced depending on addition of variables for neural induction (NIM, MNPs, and 3D; sequentially from groups 1 to 5) (Fig. 4G and H).

Consequently, undifferentiated hESCs did not show neural induction and remained pluripotent in the form of cellular colonies. On the contrary, neurally induced 2D and 3D hESCs (groups 2, 3, and 5), lost pluripotency and their differentiation represented as red fluorescence of neural induction marker proteins, which showed enhancing tendency toward group 5. Furthermore, according to red fluorescence in group 5, the morphology of cells was elongated linearly with neurites extended from each cell.

### Related mechanisms to accelerated neural induction of hEBs

To verify the related mechanisms of activated neural induction in group 5, possible signal pathways which may have been involved were suggested and investigated. Therefore, the expression of related signaling proteins was examined with immunocytochemistry and western blot. The proteins investigated for the analysis were as follows: GDNF, representing the dopaminergic neuronal pathways; NCAM, indicating cell adhesions and intercellular communications; MAP2, related to microtubule growth and neural development promoted through mechanotransduction; FAK, representing the induced mechanical stimuli [67–69].

**Fig. 5.**
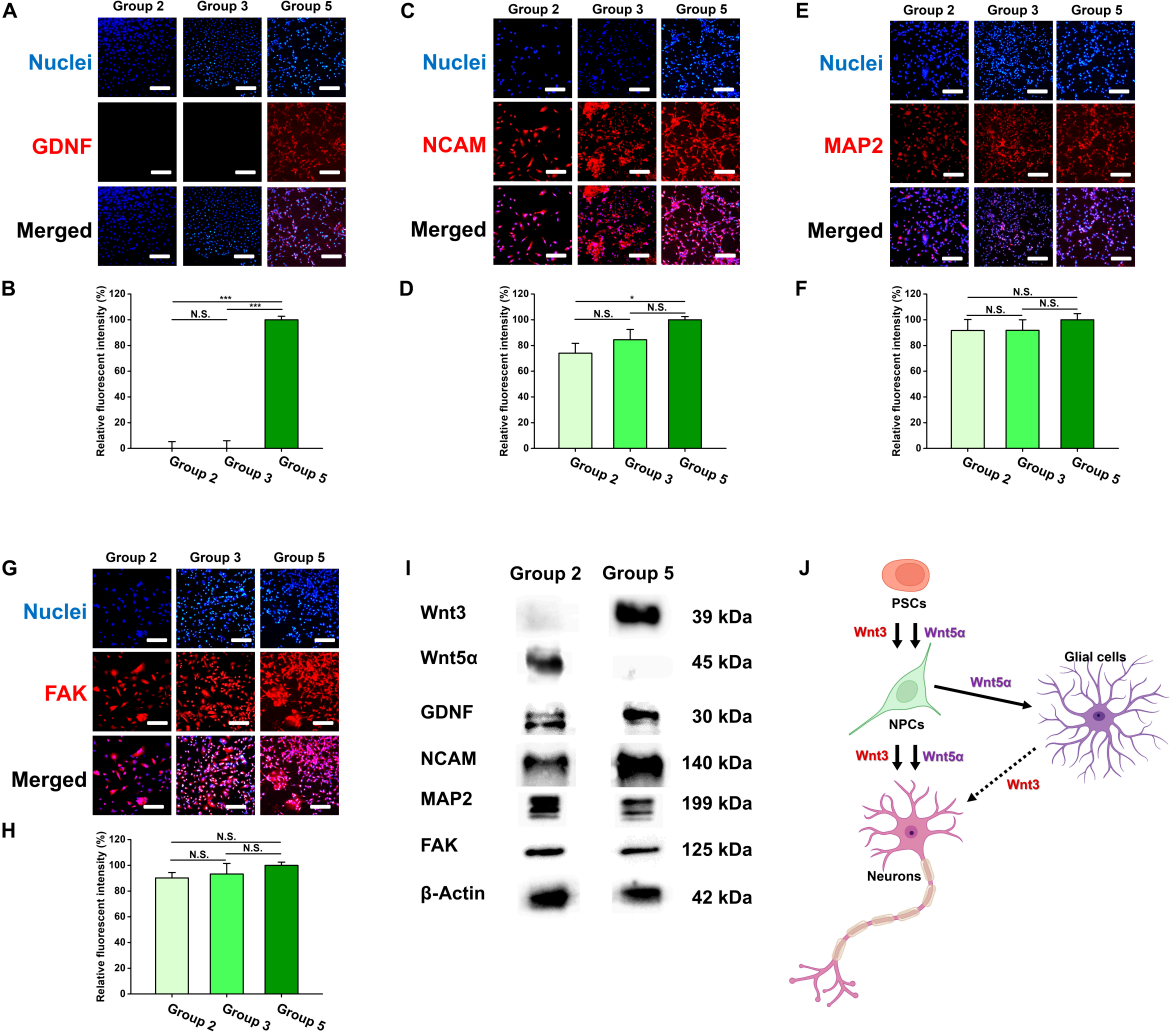
Analysis of signaling pathways of the neurally induced 2D and 3D hESCs. (A) Expression of dopaminergic neuronal pathway protein, GDNF. Nuclei were observed as blue and GDNF was shown as red. (B) Quantified fluorescence intensity of GDNF. The expression of GDNF showed a statistically significant increase in group 5, compared to the other groups. (C) Expression of intercellular communication protein, NCAM. (D) Quantified fluorescence intensity of NCAM. The expression of NCAM showed a statistically significant increase in group 5, compared with conventionally differentiated hESCs (group 2). (E) Expression of cytoskeleton-related protein, MAP2. (F) Quantified fluorescence intensity of MAP2. (G) Expression of mechanotransduction protein, FAK. (H) Quantified fluorescence intensity of FAK. All the differentiated hESCs were neurally induced with NIM for 5 d. After 5-d-long differentiation, the hEBs were also observed in a 2D attached state in order to confirm the single cellular morphology as in 2D hESCs. Therefore, cell migration from the hEBs to surroundings was also observed. (I) Western blotting of proteins related to cell signaling pathway and cell-to-cell interactions. As a reference protein, β-actin was utilized. Wnt3 was expressed only in group 5, while Wnt5α was expressed only in group 2. (J) Wnt signaling pathways involved in initial neural differentiation. The activation of Wnt3 induces conversion of pluripotent stem cells (PSCs) into neural progenitor cells (NPCs), and further differentiation of the NPCs into mature neurons. Wnt5α pathway mediates the differentiation of NPCs into neurons or glial cells represented as astrocytes. Scale bars, 200 μm. *P < 0.05, and ***P < 0.001.

In Fig. 5A, expression of GDNF was observed in groups 2, 3, and 5. Nuclei were shown as blue, while GDNF was observed as red. Different from the other groups, expression of red fluorescence was remarkably observed in group 5. In Fig. 5B, fluorescence in the image was quantified, and relative intensity values were compared among the groups. The expression level of GDNF was normalized by value of group 5, which showed maximum expression (100%). Next, the expression of NCAM was investigated in Fig. 5C and D. There was no statistical significance in the expression of NCAM between neurally induced 2D hESCs regardless of the existence of MNPs (groups 2 and 3). However, NCAM expression in group 5 showed a statistical increment compared to group 2 (*P* < 0.05). Regarding the expression of MAP2 and FAK (Fig. 5E and G), there was no statistical significance in both MAP2 and FAK expression among the groups (Fig. 5F and H).

Further, the expression of Wnt3 and Wnt5α protein was investigated through western blot with immunocytochemically examined signaling proteins (Fig. 5I). As a result, expressed Wnt3 was remarkably observed in group 5, and Wnt5α was expressed in group 2. Also, the result showed that GDNF, MAP2, and FAK were similarly expressed in both groups, while the expression of NCAM showed enhancement in group 5, compared to group 2. Therefore, neural induction-related signaling proteins were observed in group 5, in which physical stimulation was activated as much as in conventionally differentiated hESCs (group 2), the adherent cells.

## Discussion

According to the conventional approach inducing neurogenesis of hESCs, it has been taken 3 to 4 weeks in order to produce neuronal progenitor cells with neurites from the hESCs [70]. Then, about 4 to 5 more weeks were required to generate the cells finally matured into functional nerve cells. In this process, suspension culture of 3D cell aggregates, called neurosphere, was demanded, since the physical properties of the 3D cells mimic the stiffness of neural tissue [71,72]. Therefore, in this study, the hESCs were structured into 3D cell aggregates, called hEBs from the beginning of neural induction and then it was possible to reduce the time required to generate initial neuronal progenitor cells from more than 2 weeks to 5 d. For generation of hEBs and promotion of neural induction, MNP-based hEB production method was used (Fig. 1). Since there were 2 different factors, such as the addition of MNPs and 3D cultivation condition, between neurally induced hESCs (group 2) and MNP-hEBs (group 5), neurally induced MNP-hESCs (group 3) were suggested as another comparable group. And thus, the effect of the 3D on the improvement of neural induction could be investigated by comparing group 3 with group 5. Furthermore, hEBs generated without MNPs (group 4) were also suggested as another comparable group in order to investigate the effect of MNP-based hEB generation method on neural induction. And comparing group 3 with the control (hESCs; group 1) and group 2, synergic neural inductivity of the MNPs, when treated with NIM, could be detected. Throughout this study, effect of the hEB generation method using concentrated magnetic force system containing the MNPs on neural induction was analyzed by comparing the improvement of neural induction with the other groups.

To assess neural induction, morphological analysis was performed to analyze the appearance of neurally induced 2D and 3D hESCs. The formation of a star-like sharp-pointed protrusion from cells, called neurite, is a significant change in the progression of neuronal differentiation [73]. In addition, instead of forming compact colonies, enough intercellular spaces between the cells are observed depending on neural differentiation [74]. However, as neurons mature, intercellular interactions become increasingly active through neurites, which can improve cell-to-cell interactions [75]. In Fig. 2B, proportion of cells without neurites was lowest in MNP-treated groups (groups 3 and 5), while proportion of cells with more than 3 neurites was highest in group 5. As the proportion of cells without neurites decreased, the difference between the number of neurites in total cells (black bars) and cells sprouting neurites (gray bars) also decreased (Fig. 2C). According to Fig. 2C, the number of neurites per cell in cells sprouting neurites (black bars) of group 2 was higher than the values ​​of groups 3 and 4. However, this is a kind of problem that can be considered as a statistical trap. Looking at the table in Fig. 2B, in group 2, cells without neurites (number of neurites = 0) are 64.1%, and thus cells with more than 1 neurite are only 35.9%. On the other hand, in group 3, cells without neurites account for only 9.6% (less than 10%) of the total, and therefore, cells with at least one neurite exceed 90%. In other words, what is covered in “cells sprouting neurites (dark gray bar)” are only cells with actual neurites, so in group 2, the value was calculated only with cells within 35.9%. At this time, in group 2, the number of cells with 3 or more neurites is approximately 10% of the total, but within 35.9%, it is a very large value, equivalent to one-third. On the other hand, in group 3, the total cells and cells with neurites are not significantly different. Resultingly, the actual number of cells with 3 or more neurites is much higher in group 3 than in group 2 (about 3 times). Moreover, in group 5, the length of primary neurites, sum of primary and secondary neurites, and sum of total neurites were longest (Fig. 2D). In addition, the difference between the length of total neurites (blue line and table) and primary neurites (red line and table) also increased in group 5 (Fig. 2E). Therefore, it was considered that not only the length of primary and secondary neurites but also the length of shorter neurites also increased in group 5. As a result, when compared 2 3D hESCs (groups 4 and 5), significant changes in cell morphology were observed. Such difference indicated the significance of MNP-based hEB generation method on neural induction, since there were small protrusions from the cell body despite 3D suspended condition in group 4, while there were increased number of protrusions in group 5 due to enhanced cell-to-cell interaction through MNPs and magnetic force.

In genetical analysis for evaluating neural induction, representative neural induction marker genes such as *GAP43*, *TUBB3*, *NES*, and *GFAP* were investigated (Fig. 3). According to the result, the genetic expression of *GAP43*, *NES*, and *GFAP* was up-regulated in group 5. Therefore, through the expression of *GAP43* neuronal growth can be confirmed [60], and radial growth of axon could be estimated through *NES* up-regulation [9]. Of course, the expression of *GFAP* is a phenomenon that occurs not only in neuronal cells but also in astrocytes, so it is difficult to directly link the up-regulation of *GFAP* to the characteristics of either cell (neuronal cells or astrocytes). Nevertheless, it can be considered that the cells in group 5 have been undergoing neuronal differentiation, regarding genetic expression of all 3 genes [62–65]. However, only *TUBB3* showed a tendency to be expressed differently from the other genes. The expression of *TUBB3* in suspended 3D hESCs (groups 4 and 5) decreased compared to attached 2D hESCs (groups 2 and 3) in Fig. 3C. Considering that the role of *TUBB3* is related to microtubule formation [61], the 3D culture condition, in which cells floated, would down-regulate cytoskeletal production, comparing with the 2D culture condition, in which cells adhere to the bottom, generating cellular structures such as cytoskeletons [76]. However, *TUBB3* gene expression alone cannot explain all neural differentiation in terms of morphology, such as organelle development and neurite formation. According to the expression of MAP2 protein in Fig. 5E and F, it was confirmed that cells in group 5 generated and organized sufficient cytoskeletons represented as microtubules, like neurally induced 2D hESCs (groups 2 and 3). Also, actual morphology of cells in group 5 showed enhanced neurite outgrowth and morphological development in Fig. S5. Therefore, it can be inferred that although *TUBB3* genetic expression was down-regulated in group 5, the final complex of cytoskeletons was successfully produced by the interplays of various related factors [77–79].

According to the analysis of neuronal protein expression through immunostaining, similar tendency was observed with the gene expression results (Fig. 4). The protein expression of GFAP, PAX6, and PROX1 was also significantly higher in group 5. This provides another evidence for neuronal development, considering that PAX6 supports ectodermal differentiation, and PROX1 is a factor involved in hippocampal formation [66]. In addition, group 3 showed statistically improved neural induction compared to groups 1 and 2. In our previous study regarding spontaneous differentiation of the hEBs generated using the concentrated magnetic force and the MNPs, the MNPs themselves had no significant effect in directing hESC fate [17]. However, according to our results in this study, when MNPs were applied with differentiation conditions, as in group 3, MNPs appeared to induce or aid differentiation compared with non-MNPs conditions, as in group 2. Resultingly, although MNPs themselves do not regulate the differentiation of hESCs [17], MNPs can synergistically affect specific differentiation when treated with differentiation medium such as neural induction media. In this study, hEBs were observed after attached into 2D state, since the 5-d-long hEBs were not a compact tissue but a loosened spheroid, which contributed failure of cross sectioning. For improved understanding of neural differentiation of the hEBs, differentiation and apoptosis of the core cells in hEBs have to be investigated through an advanced strategy generating more organized hEB forms by long-term culture.

To find the cause of enhanced neural induction in group 5, relative signaling pathways were analyzed (Fig. 5). The protein level expression of NCAM, MAP2, and FAK was observed in 3 neurally induced groups (groups 2, 3, and 5). Through this, an increase in the expression of NCAM, microtubule-associated protein, and focal adhesion kinase was confirmed, respectively. In particular, in the case of group 5, the expression of GDNF also significantly increased, compared with the other groups. This can be understood to mean that cells in group 5 have capability for dopamine affinity [67–69]. Furthermore, in Fig. 5I, Wnt signaling pathways were investigated through western blot of Wnt3 and Wnt5α proteins. Although both of Wnt proteins are known to be involved in initial neural differentiation of pluripotent stem cells (PSCs) to neural progenitor cells (NPCs), the exact function of each protein is different [80–83] (Fig. 5J). Related studies have shown that activation of Wnt3 not only induces PSCs into NPCs but also results in the differentiation of NPCs into mature neurons, whereas Wnt5α mediates the differentiation of NPCs into neurons and glial cells [84–86]. Therefore, it is believed that group 5 was induced to differentiate into the neuronal lineage rather than the glial lineage by the Wnt3 signaling pathway, not Wnt5α pathway [80–83]. Consequently, cells in group 5 followed Wnt3 signaling pathway and showed dopamine affinity mediated by the increased expression of NCAM, MAP2, and FAK. Also, the enhanced cell-to-cell interactions by the magnetic stimuli were considered to be a driving force for the enhancement of initial neural induction.

As the overall experiments progressed, the number of experimental groups decreased, changing the number of experimental groups for each figure. We first set undifferentiated hESCs (group 1) and conventionally differentiated hESCs (group 2) as controls for MNP-hEBs (group 5). However, since there are 2 differences between groups 2 and 5, presence of MNPs and 3D condition, we also set group 3 (group 2 + MNPs) and group 4 (group 2 + 3D). As a result, 5 experimental groups such as group 1 to 5 were initially established, and thus cellular morphology analysis (Fig. 2) and genetic analysis (Fig. 3) were conducted using these 5 groups. According to the results in both Figs. 2 and 3, the group with the next highest value following group 5 was groups 2 and 3. The induction of early neural differentiation is interpreted to be more influenced by the effect of MNPs than by the effect of 3D. Therefore, groups 2 and 3 were considered to be the practical control groups comparable to group 5, and for this reason, group 4 was excluded from subsequent experiments. As a result, protein expression analysis (Fig. 4) was performed in 4 experimental groups (groups 1, 2, 3, and 5). Finally, in Fig. 5, mechanotransduction and signaling pathways were analyzed. As the need for an undifferentiated group in which no neural induction occurred at all disappeared, the experimental groups were ultimately set as 3 groups (groups 2, 3, and 5). Further, since no significant difference was observed between groups 2 and 3 in protein level analysis, in the last analysis through Western blotting in Fig. 5I, group 2, the initial experimental control group of group 5, was used as originally intended.

For clinical applications of our study, using MNPs-incorporated stem cells, there are still several issues that need to be addressed. If the hESCs incorporated with MNPs are to be transplanted and then used for neural tissue regeneration, it is necessary to first confirm the clinical applicability of the MNPs. According to the cell viability assay performed in our former work to confirm the biocompatibility of MNPs, no cytotoxicity was observed even when treated at a concentration of 50 μg/ml, which was more than twice higher compared to the 20 μg/ml used in this study [17]. Also, the amount of MNPs introduced in a cell was measured [49]. When 20 μg/ml of the MNPs were applied to mesenchymal stem cells (MSCs), about 160 pg of MNPs was accumulated in a MSC. Considering that the average diameter of MSCs is about 20 μm, which is twice as large as the hESCs, the amount of MNPs introduced per hESC can be expected to be about 20 pg (one-eighth MNPs in a MSC as a relative to the volume ratio). However, as shown in Fig. S2, MNPs remain accumulated in the intracellular vesicles rather than spread throughout the cytoplasm. Therefore, the exact amount of introduced MNPs per a hESC could be different from the estimated value. In addition, there has been decrease in the concentration of MNPs in a cell over time [49]. From day 10, the percentage of magnetized cells relative to total cells decreased by about 30%, which is considered to be due to the dilution effect of MNPs caused by cell proliferation. In addition, according to Fig. S2, the MNPs exist in the endosomal structures of the cells instead of being dispersed in cytosols. Assuming through the distribution pattern of the intracellularly delivered MNPs, degradation of the MNPs under low-pH conditions in endosomes could be another factor of the MNPs dilution [87]. Further, the detailed mechanisms related to the synergic effect of the MNPs on neural induction should be revealed, and thus, the exact role of the MNPs on neural induction of the hESCs has to be established. According to the results, it was confirmed that the 3D condition alone did not show a significant difference in inducing early neural differentiation. On the other hand, the MNPs showed clear contribution to enhanced initial neural differentiation through group 3. Regarding there are only 2 groups in which MNPs are applied (groups 3 and 5), MNPs are considered to have an obvious effect on neural differentiation. Therefore, MNPs by themselves can cause significant differences in initial neural induction when applied with differentiation condition such as differentiation medium, while 3D conditions alone cannot have a significant effect on the induction of early neural differentiation. Nevertheless, 3D conditions can affect early-stage neural differentiation in a synergic direction when preceded by the presence of MNPs. Also, it is necessary to investigate the changes in intercellular communication and subsequent signals in neurally induced hESCs. In order to confirm the functional advance, long-term differentiation and maturation is required. Through related studies regarding the effect on mechanotransduction on electrophysiological functionality, physical stimuli have a significant effect on neural maturation and generation of nerve cells with electrofunctionality [88–91]. In our future work, this study could be applied for generation of functionally mature nerve cell, and thus, expression of not only late-stage markers but also synaptic markers should be analyzed. Also, through follow-up research in which long-term differentiation conducted achieving complete neuronal differentiation and generating functionally perfect nerve cells, practical applicability of this research results should be explored through in vivo experiments.

Nevertheless, this study not only suggested an efficient neuronal induction method but also revealed the significant factors for initial neural differentiation: hEB generation in uniform size, and enhanced cell-to-cell interactions through MNPs. Therefore, MNP-based hEB generation could be an alternative to neuronal tissue regeneration, supporting to understand hESC commitments during the initial embryogenesis.

Although there have been many efforts to regenerate the nerve tissue by differentiating the pluripotent stem cells into neuronal cells, the neural lineage specification requires long-term cultivation and laborious steps. In this study, MNP-incorporated and uniformly sized hEBs were generated for improved neural differentiation. The neural inductivity was compared in neurally induced MNP-hEBs (group 5) with the other experimental groups in order to define the effect of each variable for neural differentiation (NIM, MNPs, and 3D): hESCs (group 1); conventionally differentiated hESCs (group 2); neurally induced MNP-hESCs (group 3); neurally induced hEBs (group 4). According to the results, group 5 showed improved initial neural differentiation in morphological analysis, genetical investigation, immunocytochemistry, and western blotting. Furthermore, neurally induced MNP-hEBs in group 5 followed Wnt3 signaling pathway and possessed dopamine affinity and enhanced cell-to-cell interactions. Therefore, MNP-based hEB size control method proposed in this study would be a useful tool for enhanced neuronal differentiation and nerve tissue regeneration. In addition, this technique could be applied to accelerate initial lineage-specific differentiation of hESCs, directing cellular commitments with simulation of embryogenesis.

## Data Availability

The data that support the findings of this study are available from the corresponding author upon reasonable request.
