# Supplementary material for: Improved Neural Inductivity of Size-Controlled 3D Human Embryonic Stem Cells Using Magnetic Nanoparticles
Source: Biomater Res. 2024 Mar 15;28:0011. doi: 10.34133/bmr.0011 (PMC10944702; doi:10.34133/bmr.0011)
Supplement: Supplementary 1 — Figs. S1 to S7 [file bmr.0011.f1.docx]

***Biomaterials Research***

**Supplementary Information**

**Improved Neural Inductivity of Size-Controlled 3D Human Embryonic Stem Cells Using Magnetic Nanoparticles**

Boram Son^1,2,#^, Sora Park^1,#^, Sungwoo Cho^1^, Jeong Ah Kim^3^, Seung-Ho Baek^4^, Ki Hyun Yoo^5^, Dongoh Han^5^, Jinmyoung Joo^6^, Hee Ho Park^2,7,^*, and Tai Hyun Park^1,8^*

^1^School of Chemical and Biological Engineering, Institute of Chemical Processes, Seoul National University, 1 Gwanak-ro, Gwanak-gu, Seoul 08826, Republic of Korea

^2^Department of Bioengineering, Hanyang University, 222 Wangsimri-ro, Seongdong-gu, Seoul 04763, Republic of Korea

^3^Center for Scientific Instrumentation, Korea Basic Science Institute, Cheongju, Chungbuk 28119, Republic of Korea

^4^Center for Bio-based Chemistry, Korea Research Institute of Chemical Technology (KRICT), Ulsan, 44429, Korea

^5^SIMPLE Planet Inc., 48 Achasan-ro 17-gil, Seongdong-gu, Seoul, 04799, Korea

^6^Department of Biomedical Engineering, Ulsan National Institute of Science and Technology (UNIST), Ulsan 44919, Republic of Korea

^7^Research Institute for Convergence of Basic Science, Hanyang University, Seoul 04763, Republic of Korea

^8^Department of Nutritional Science and Food Management, Ewha Womans University, Seodaemun-gu, Seoul, 03760 Republic of Korea

^#^ These authors contributed equally.

* Correspondence to [parkhh@hanyang.ac.kr](mailto:parkhh@hanyang.ac.kr) and [thpark@ewha.ac.kr](mailto:thpark@ewha.ac.kr)


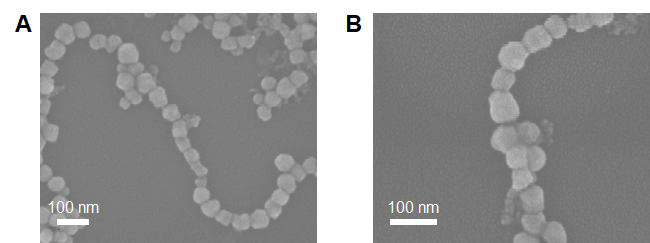


**Supplementary Fig. S1 Morphology of MNPs.** MNPs dispersed in PBS were observed through SEM. Scale bars indicate 100 nm.


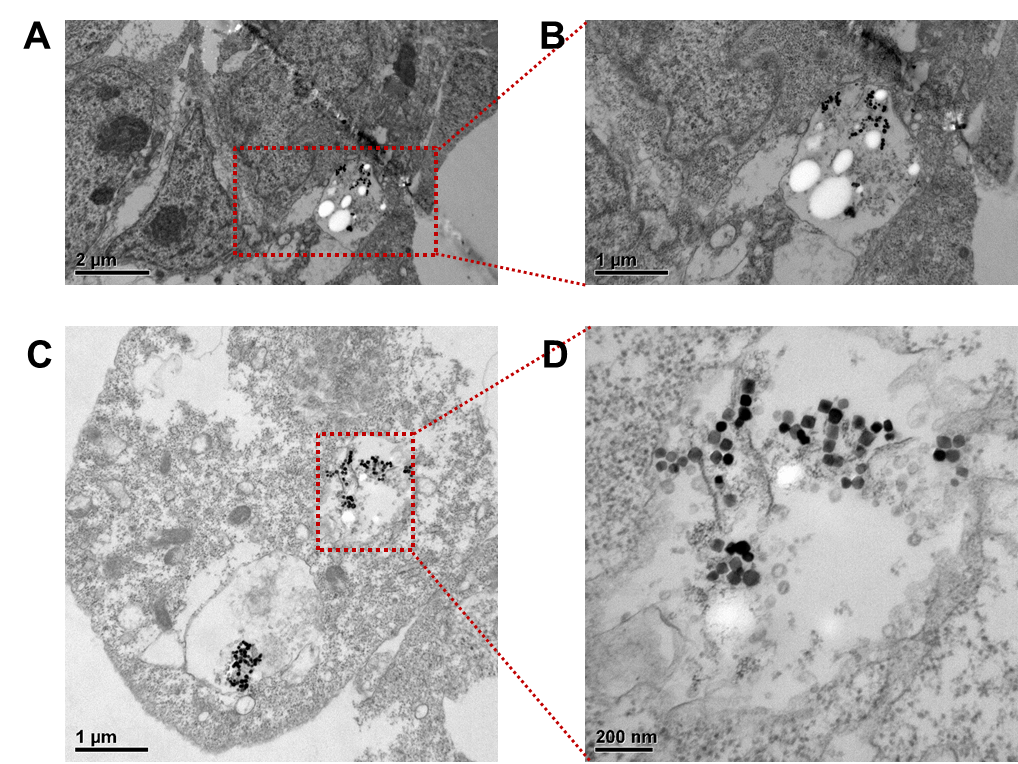


**Supplementary Fig. S2 Morphology of the intracellularly delivered MNPs.** The MNPs incorporated into hESCs were observed through TEM. Intracellularly delivered MNPs were encapsulated by endosomic structures. Enlarged images of the red squares in (A) and (C) are (B) and (D), respectively. Scale bar in (A) indicates 2 μm. Scale bars in (B) and (C) indicate 1 μm, respectively. Scale bar in (D) indicates 200 nm.

**
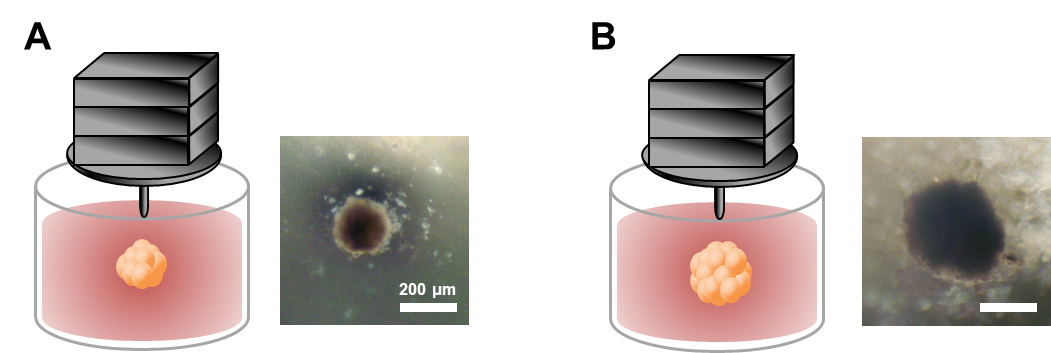
**

**Supplementary Fig. S3 Morphology of the small and large EBs.** The 3D EBs were generated using MNPs and a concentrated magnetic force system. For small EB (150 μm in diameter) generation, 10,000 cells were applied to a well of 96 well-plates. And for large EB (600 μm in diameter) generation, 160,000 cells were added to a well. All the cells used in EB formation were treated with the MNPs, followed by isolation via static magnets. Scale bars indicate 200 μm.


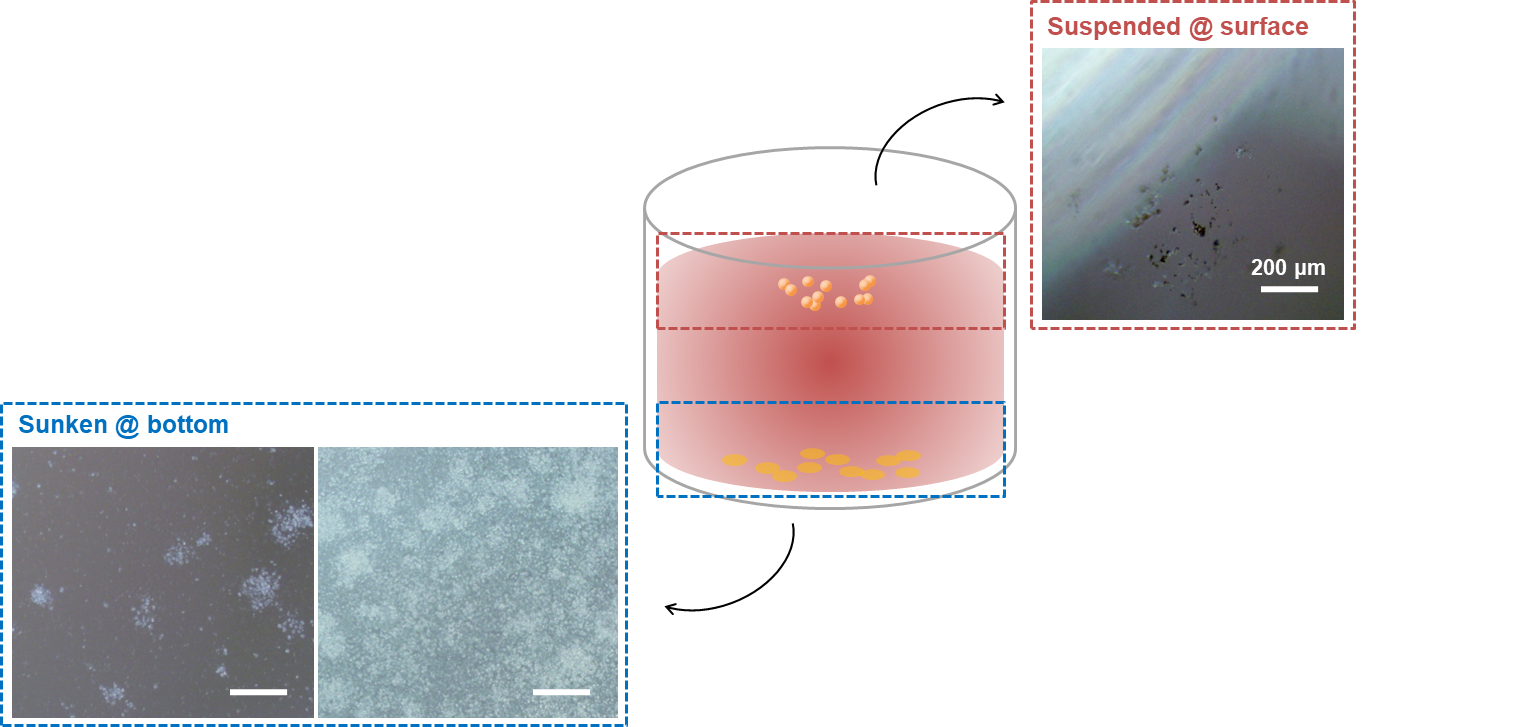


**Supplementary Fig. S4 hESCs on surface and bottom without concentrated magnetic force system.** When the magnetized hESCs were added to a well of 96-well plates without the concentrated magnetic force system, some of the cells were suspended on surface and the others were sunken on the bottom, resulting in failure of hEB formation. Scale bars indicate 200 μm.


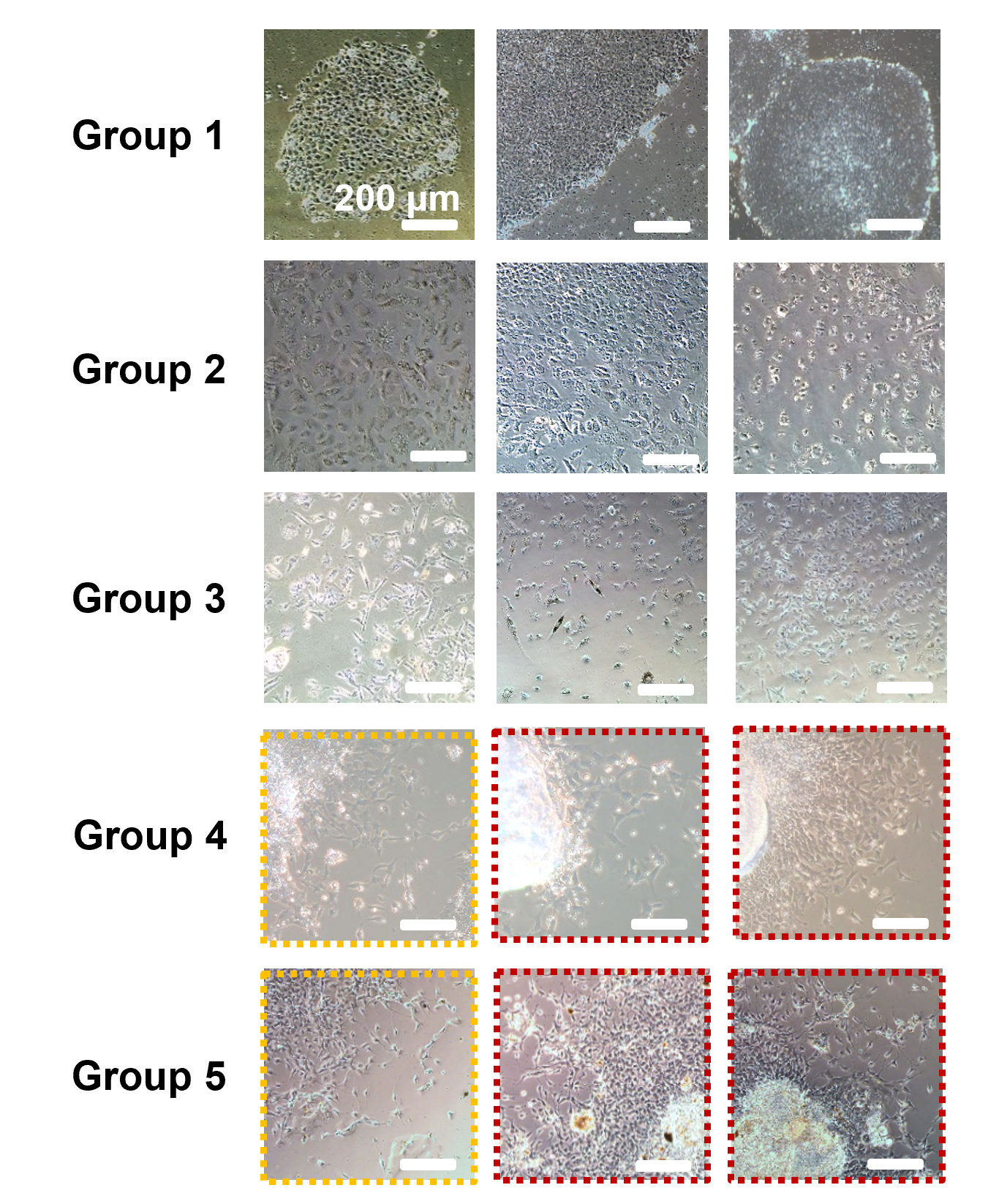


**Supplementary Fig. S5 Magnified photographs of the differentiated 2D and 3D hESCs.** Microscopic images of hESCs in **group 1 to 5** are enlarged from **Fig. 2A**. All the differentiated hESCs were neurally induced with NIM for 5 days (**group 2 to 5**). After 5 day-long differentiation, the hEBs were also observed in a 2D attached state in order to confirm the single cellular morphology as in 2D hESCs. Therefore, cell migration from the hEBs to surroundings was also observed. Yellow dotted squares indicate migrated cells from the 3D aggregates. Red dotted squares indicate the edge of hEBs, including 3D aggregates and migrated cells. Scale bars, 200 μm.


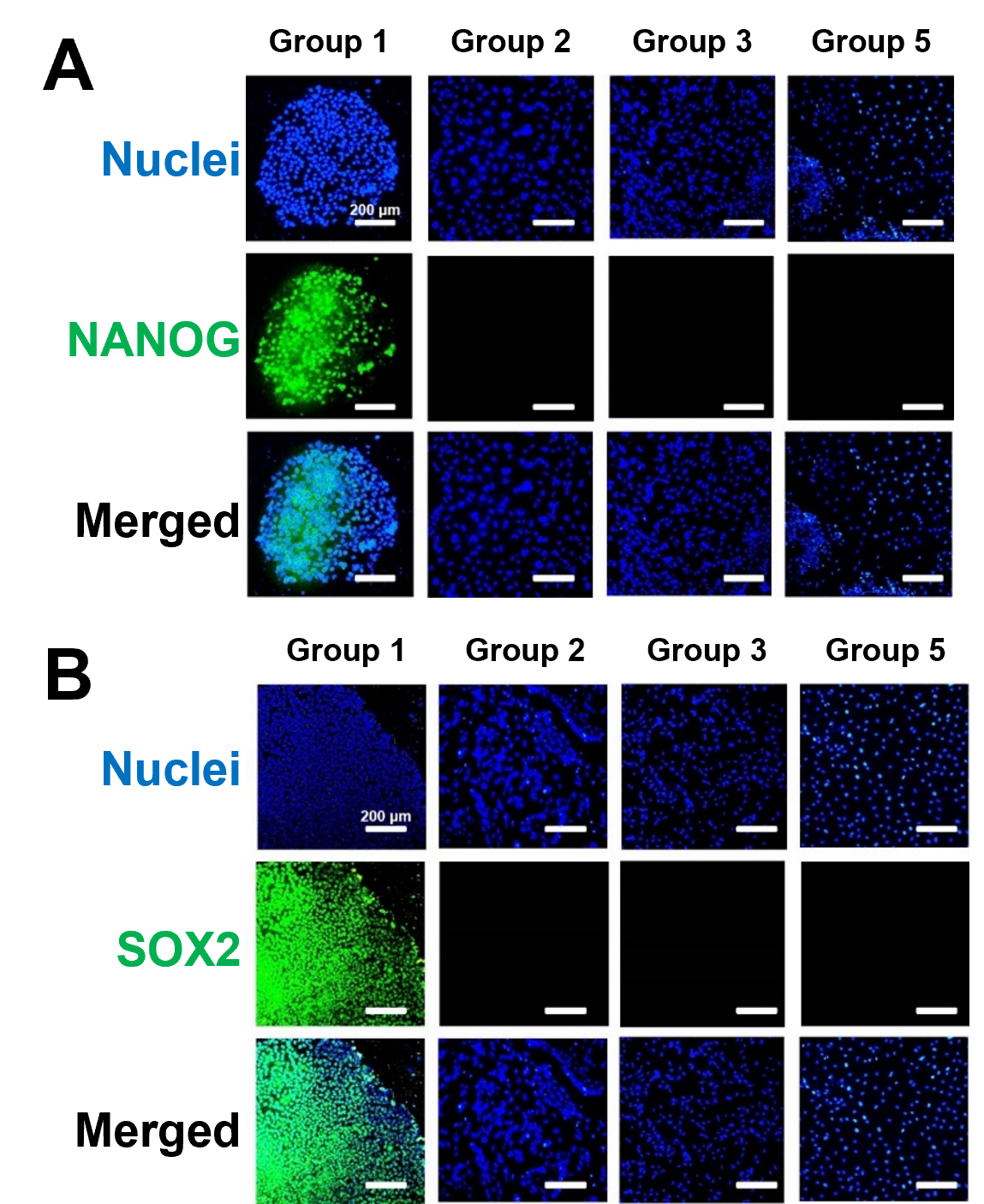


**
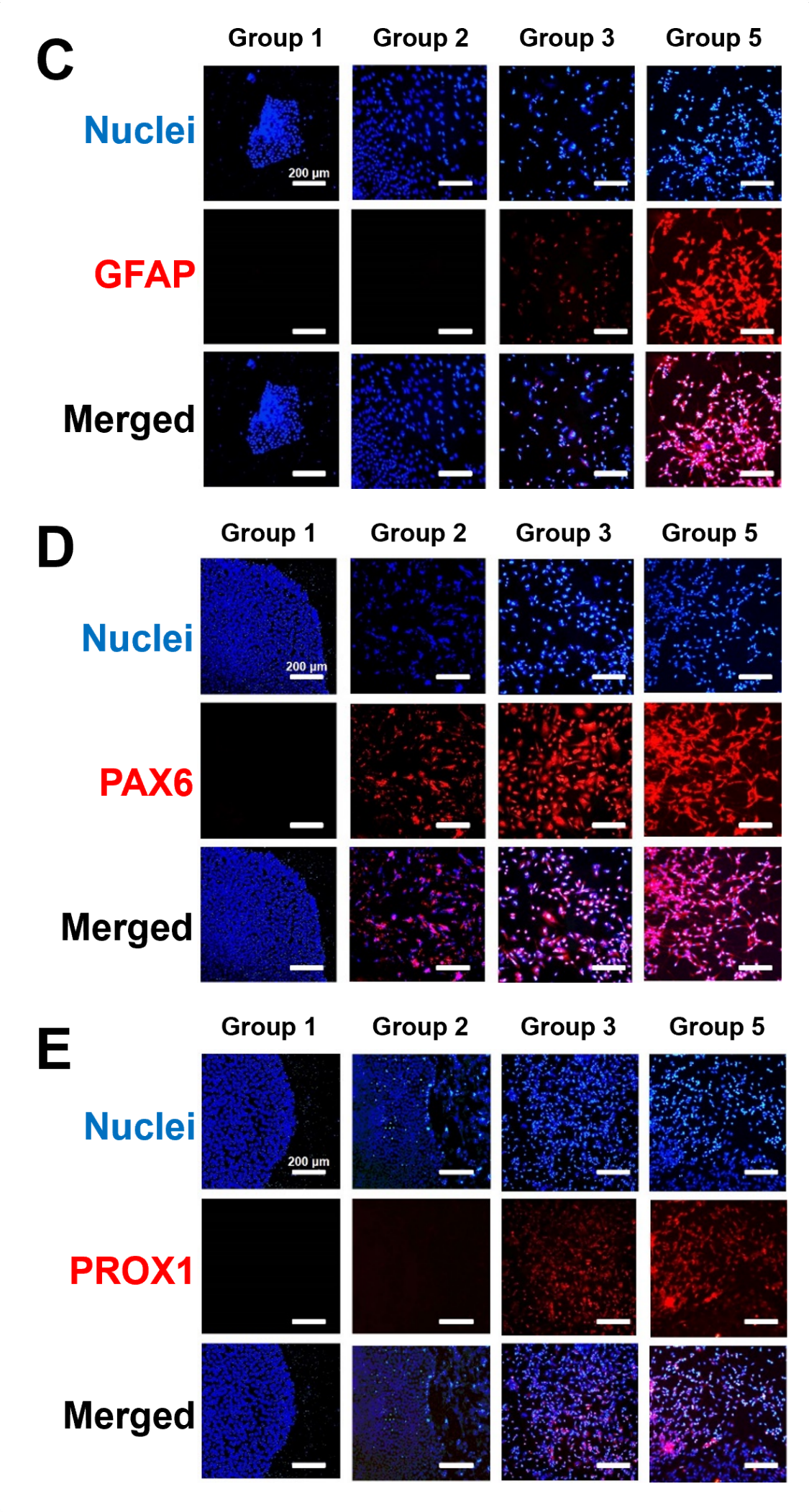
**

**Supplementary Fig. S6 Magnified immunocytochemical photographs of the differentiated 2D and 3D hESCs.** Immunocytochemical images of hESCs in **group 1, 2, 3, and 5** are enlarged from **Fig. 4**. Nuclei were presented as blue, pluripotency marker proteins such as NANOG (A) and SOX2 (B) were shown as green, and neuronal marker proteins such as GFAP (C), PAX6 (D), and PROX1 (E) were represented as red. Scale bars, 200 μm.


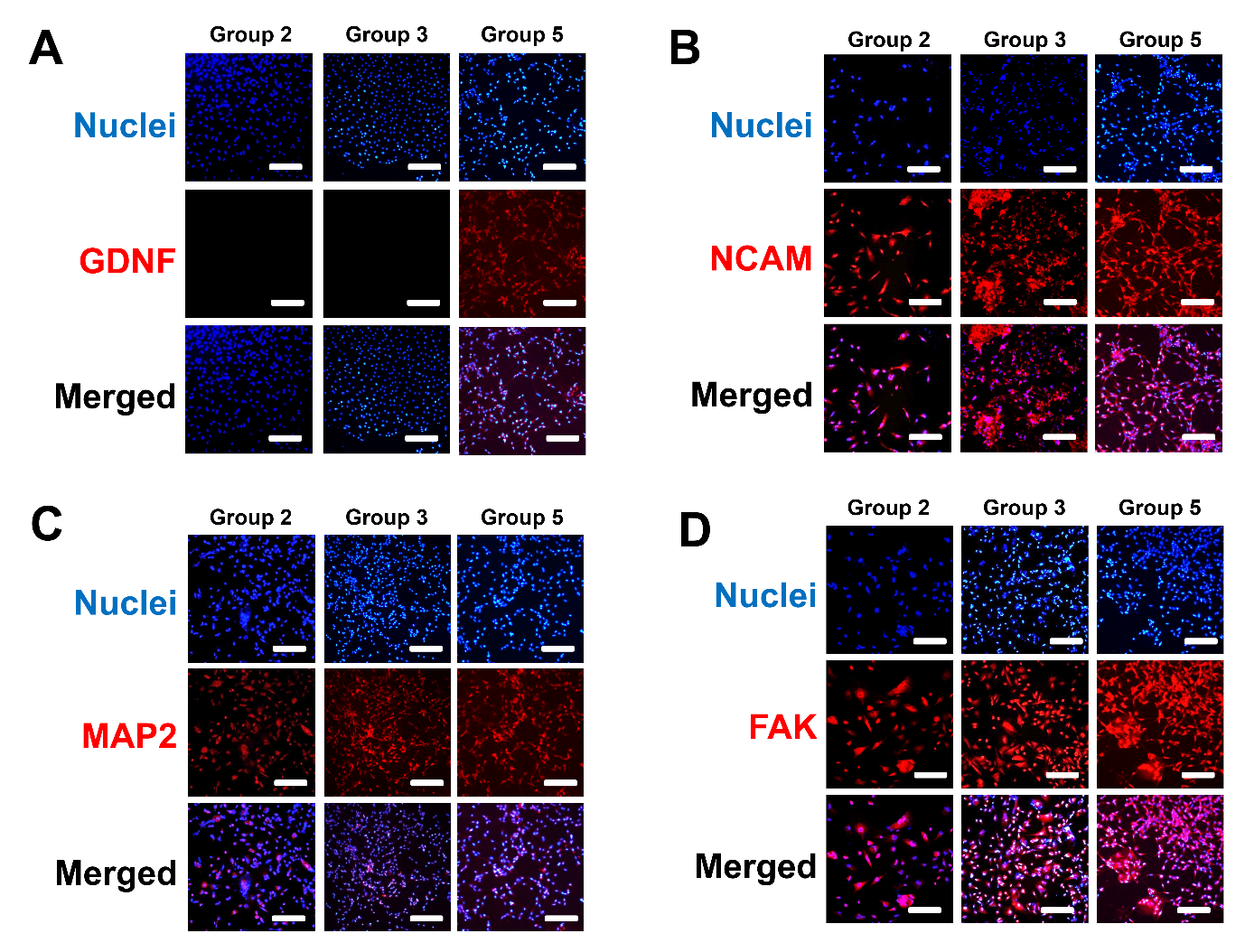


**Supplementary Fig. S7 Magnified immunocytochemical photographs of the differentiated 2D and 3D hESCs.** Immunocytochemical images of hESCs in **group 2, 3, and 5** are enlarged from **Fig. 5**. Nuclei were presented as blue. GDNF (A), NCAM (B), MAP2 (C) and FAK (D) were represented as red. Scale bars, 200 μm.
